# Height and Body Composition Determine Arm Propulsive Force in Youth Swimmers Independent of a Maturation Stage

**DOI:** 10.2478/hukin-2014-0081

**Published:** 2014-10-10

**Authors:** Tatiane Moura, Manoel Costa, Saulo Oliveira, Marcos Barbosa Júnior, Raphael Ritti-Dias, Marcos Santos

**Affiliations:** 1 Human Performance Evaluation Laboratory, Faculty of Physical Education, University of Pernambuco (LAPH/ESEF/UPE), Pernambuco, Brazil.; 2 School of Physical Education, University of Pernambuco, Pernambuco, Brazil.; 3 Anthropometrist level 1 (Certification by ISAK).; 4 Department of Physical Education and Sports Science, Federal University of Pernambuco, Academic Center of Vitória de Santo Antão, Pernambuco, Brazil.

**Keywords:** body composition, performance, swimming, anthropometric variables, propulsive force

## Abstract

The aim of this study was to examine the relationship between anthropometric variables, body composition and propulsive force in swimmers aged 9–17 years. Anthropometric characteristics (body height and mass, sitting height, arm span, arm muscle area and body composition) and the propulsive force of the arm (tethered swimming test) were evaluated in 56 competitive male swimmers. Tanner’s stages of genital maturation (P1–5) were used. The data analysis included correlations and multiple linear regression. The propulsive force of the arm was correlated with body height (r = 0.34; p =0.013), arm span (r = 0.29; p =0.042), sitting height (r = 0.36; p =0.009), % body fat (r = 0.33; p =0.016), lean body mass (r = 0.34; p =0.015) and arm muscle area (r = 0.31; p =0.026). Using multiple linear regression models, the percent body fat and height were identified as significant predictors of the propulsive force of the arm after controlling for the maturation stage. This model explained 22% (R2 = 0.22) of associations. In conclusion, the propulsive force of swimmers was related to body height and percent body fat.

## Introduction

Swimming involves generation of propulsive forces sufficient to overcome the effects of water resistance on the body. Thus, the quality of a swimmer’s performance is influenced by his or her ability to generate such a force at a satisfactory level (Schneider and Meyer, 2005). The ability to increase propulsive forces improves with technique, biomechanical parameters and physical conditioning of the swimmer. Moreover, the swimmer’s physical condition is related to body composition and strength (Schneider and Meyer, 2005). Therefore, certain anthropometric characteristics must be considered when analysing the performance of swimmers in view of the relationship between the resistive and propulsive forces (Santos et al., 2012).

Because both morphological characteristics and metabolic capacities are affected by growth and development, the influence of those factors on swimming performance may vary between youth and adult swimmers (Latt et al., 2009a). Several studies have examined the relationship between anthropometric characteristics and performance of swimmers (Geladas et al., 2005; Jurimae et al., 2007). Geladas et al. (2005) found that the total length of the upper limbs, grip strength and standing long jump performance were significant predictors of performance in 100-meter front crawl races in boys (12–14 years old). Another study found a positive association (*r* = 0.68, p < 0.001) between the arm muscle area and the propulsive force of the arm in young swimmers (9–14 years old), with the increased arm muscle area contributing to a greater capacity for strength (Santos et al., 2012). However, it is important to highlight that because the maturation stage has a direct influence on a child’s natural growth by increasing the levels of circulating hormones, the maturation stage is a key factor that must be controlled when studying children and adolescents (Geladas et al., 2005).

One previous study followed 29 female swimmers for a two-year period. It was observed that swimming performance at different stages of maturation was related primarily to body size (height) and arm span, thereby reflecting a higher VO_2peak_ (ml/kg·min^−1^) and improved biomechanical swimming variables (Latt et al., 2009b). In this context, Caputo et al. (2006) reported that for events with a short duration, in which the power production capacity is considered a key variable, physical characteristics such as body height, arm span, body composition and somatotype can also contribute to the level of performance (Caputo et al., 2006). However, these morphological attributes largely depend on genetic factors and may have a decisive influence on swimming performance (Latt et al., 2009b).

Data on the relationships between anthropometric characteristics and the propulsive force of youth swimmers are scarce and inconclusive, which limits comparisons. In some studies of athletes, there was no assessment considering the maturation stage of subjects. To the best of our knowledge, no studies have investigated the influence of anthropometric characteristics and body composition on the propulsive force of the arm in youth swimmers taking into account their sexual maturation. Thus, the objectives of this study were as follows: (1) identify the relationship between anthropometric characteristics and the propulsive force of the arm, and (2) determine the relative contributions of covariates such as body height, arm span, sitting height, body fat content, lean body mass and arm muscle area on the propulsive force generated during the front crawl stroke in youth swimmers.

## Material and Methods

### Participants

An appropriate sample size was estimated using G*Power software v. 3.0.10 (Franz Faul, University of Kiel, Kiel, Germany) (Faul et al., 2007) given the following conditions: effect size= 0.54; minimum power= 0.95; and α= 5%. Fifty-six male youth swimmers (14 ± 1.8 years old) registered with the Brazilian Federation of Aquatic Sports who trained on average two hours per session, six times per week, were included in this study. Each subject visited the laboratory accompanied by a researcher, who was responsible for the following: (1) analysis and clarification, (2) anthropometric measurements, and (3) evaluation of propulsive force. Informed consent was obtained from each parent or legal guardian before the start of data collection. Acceptance by the participating children and adolescents was required for participation in this study. The biological age of each swimmer was evaluated according to the method proposed by Tanner and Whitehouse (1976), which is based on self-assessment of the development of the genitals and pubic hair.

This study was approved by the Ethics Committee of the University of Pernambuco (protocol number 048/09), in accordance with the ethical standards laid down in the 1964 Declaration of Helsinki (www.wma.net/e/policy/b3.htm).

### Measures

*Anthropometric variables and body composition:* Anthropometric measurements were taken according to the methods of Lohman (1986). Standing height (cm) was measured without shoes to the nearest 0.1 cm with a stadiometer (Sanny, São Paulo, Brazil). Body mass (kg) without shoes and with light clothing was measured with a scale (Filizola, São Paulo, Brasil) to the nearest 100 g (Lohman, 1986). Sitting height was obtained with the participant sitting on an adjustable-height chair at a seat height of 50 cm. The arm span was obtained to the nearest 0.1 cm using a tape measure (Starrett, Itu, Brazil) with the individual standing with the arms abducted with a 90° angle with the trunk, with the elbows extended and the forearms supinated. The distance between the 3^rd^ finger of the right and left hand in this position was taken as the arm span (Lohman, 1986).

The relaxed arm circumference was measured by a single evaluator with a flexible tape measure with precision of 0.1 cm according to the conventional techniques described by Callaway (1991). The arm muscle area (AMA) was calculated on the basis of the equation proposed by Frisancho (1984): AMA (cm^2^) = {[AC (cm) - π · TRT (cm)] 2/4 · π, where AMA was the relaxed AMA, AC was the arm circumference, TRT was the triceps skinfold thickness, and π = 3.1416. The triceps and subscapular skinfold thickness were measured with a Lange calliper (Santa Cruz, CA, United States). The % body fat, fat mass (kg) and lean body mass (kg) were estimated using the Slaughter et al. (1988) equation for boys. Each of the previously described measurements was taken twice, and the mean value was reported. When a difference of more than 3% was observed between the two measurements, a third measurement was taken.

*Data quality control:* The data quality was assessed by retesting 10% of the total sample. Intraclass correlation coefficients (*R*) and the respective (95%) confidence intervals were used to estimate reliability, the values of which were as follows: body mass: *R* = 0.99 (95% CI: 0.98 to 1.00), body height: *R* = 0.99 (95% CI: 0.98 to 1.00), sitting height: *R* = 0.99 (95% CI: 0.98 to 1.00), arm span: *R* = 0.98 (95% CI: 0.97 to 0.99), right arm circumference: *R* = 0.99 (95% CI: 0.99 to 1.00), triceps skinfold thickness: *R* = 0.95 (95% CI: 0.87 to 0.98), and subscapular skinfold thickness: *R* = 0.99 (95% CI: 0.97 to 0.99).

### Procedures

**Figure 1:**
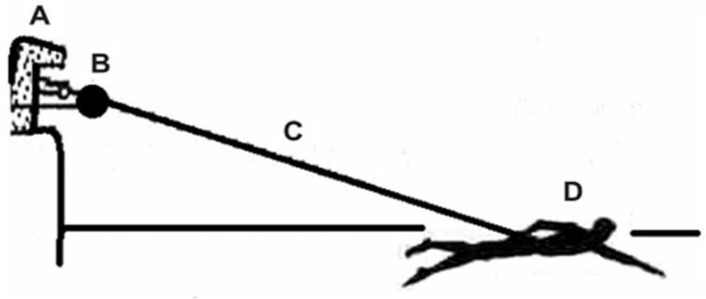
Schematic representation of the system used to determine the propulsive force of swimmer’s arms. Abbreviations: A, starting block; B, dynamometer; C, mild steel wire; D, swimmer

*Propulsive force of the arm (PFA):* The swimmers were not allowed to exercise for 24 hours before the tests to prevent the acute effects of training on results. A 10 min warm-up with moderate intensity was performed before the beginning of the tests according to previous studies (Papoti et al., 2007; Santos et al., 2012). After the swimmer had warmed up, a pull buoy or leg float was placed between the legs of the swimmer to prevent him or her from performing movements with his or her lower limbs. The tethered swimming test consisted of two 30 s periods of maximal effort during which the swimmer performed the front crawl while tied to the measurement apparatus. We used the peak force exerted between two trials for each swimmer. To determine the propulsive force the better of the two results was considered. The beginning and end of the test were marked by an audible signal (whistle), and all participants were verbally encouraged to maintain maximum speed (Papoti et al., 2007). Measurements for each athlete were obtained using a load cell with a traction capacity of 300 kg (Globus Ergometer, Codigné, Italy). This equipment was attached at the edge of the pool and fixed to the swimmer’s body (Figure 1).

### Analysis

The normality and equality of variance of the distributions were analysed using the Shapiro–Wilk test and the Levene test, respectively. Descriptive statistics are presented as the means and standard deviations and minimum and maximum values. After the correlation results (Pearson’s product-moment coefficient) for the main study variables had been evaluated, the effects of the variables on the propulsive force of the arm were analysed by multiple linear regression in an extended-model approach. Thus, a series of multiple linear regression models was consecutively tested. Separate models were built for each anthropometric characteristic and body composition variable to determine the relationship of each variable with the propulsive force of the arm. A p-value ≤ 0.05 was considered statistically significant. SPSS for Windows version 18.0 (SPSS Inc.; Chicago, IL) was used for all analyses.

## Results

Descriptive statistics for the anthropometric variables, body composition and propulsive force of the arm are shown in Table 1.

**Figure 2:**
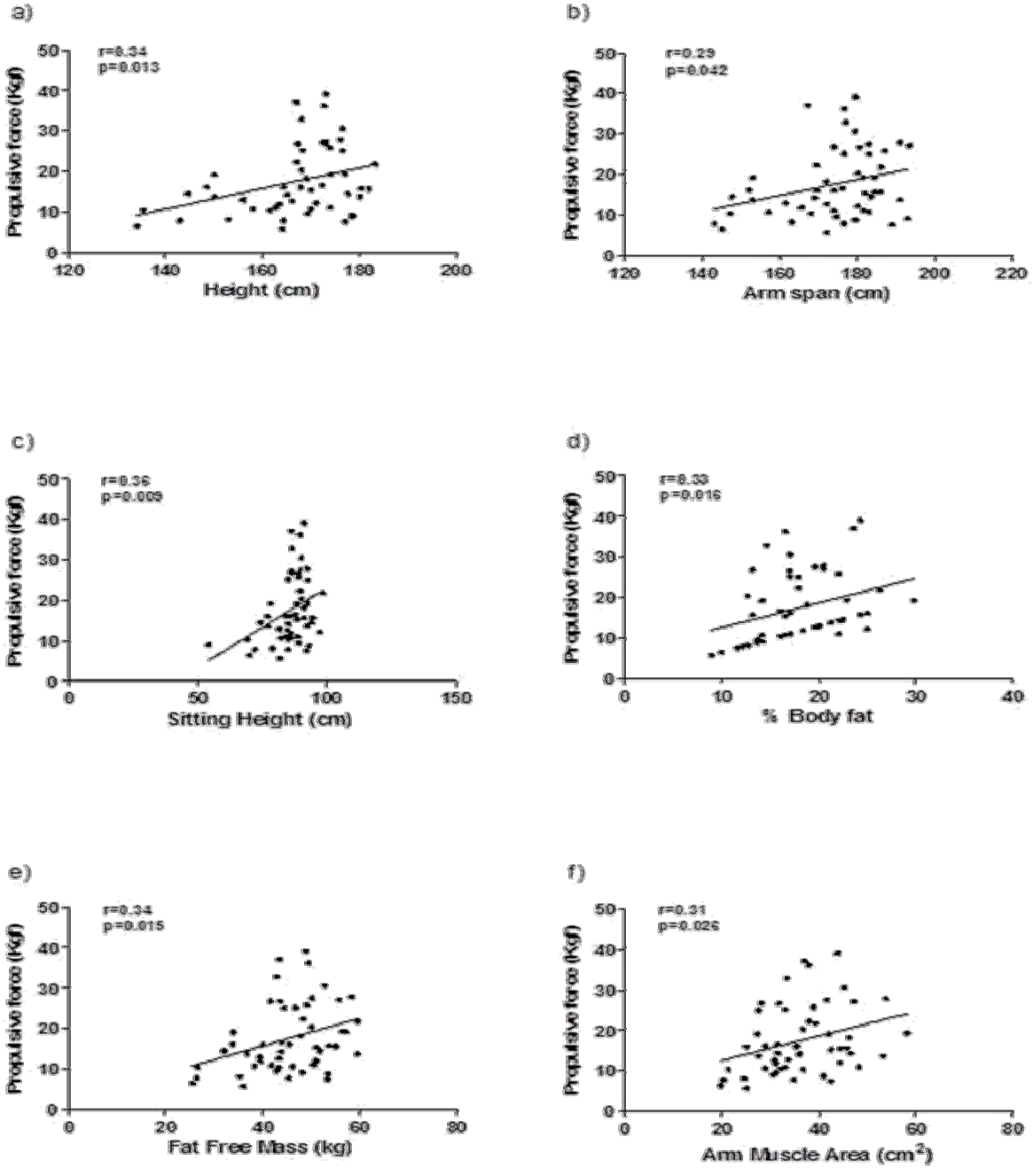
The product-moment coefficient and p-values among propulsive force of the arm, anthropometric characteristics and body composition in youth swimmers (N=56)

Figure 2 presents the Pearson correlation coefficients (r and p-values). The propulsive force was significantly positively correlated with body height (p = 0.013), arm span (p = 0.042), sitting height (p = 0.009), % body fat (p = 0.016), fat free mass (p = 0.015) and AMA (p = 0.026).

The linear regression analysis showed that the % body fat content and body height contributed significantly to the variation in arm propulsive force, after controlling for the maturation stage (*R*: 0.46). This model explained 22% (*R*^2^ = 0.22) of the variation (Table 2).

## Discussion

The present study revealed significant relationships between anthropometric variables and propulsive force of the arm during freestyle swimming. Interestingly, these relationships remained significant after controlling for the maturational stage.

Our findings corroborate with those of previous studies that found an association between body segments and the propulsive force of the arm. For example, Costa et al. (2006) used the ratio between arm span and body height as an index for identification of a swimmer’s performance. In that study, the greatest ratio corresponded to the greatest absolute speed and traction force during swimming (Costa et al., 2006). Geladas et al. (2005), who studied 263 competitive swimmers of both sexes (12 to 14 years old), found negative associations between both anthropometric variables and physical conditioning and performance in a 100 m freestyle race (*r* = − 0.46 a − 0.73; p < 0.001). Perez et al. (2011) also found significant correlations between the average swimming speed and other variables: arm length (r=0.71; p <0.05), forearm (r=0.43; p>0.05) and hand length (r=0.51; p<0.05), arm span (r=0.54; p<0.05) and biacromial diameter (r=0.45; p<0.05).

In this study, the sitting height was significantly correlated with the propulsive force of the arm, indicating that a major trunk length is related with high-performance swimming. This correlation was examined in a study using a multivariate analysis, which found that the trunk-cephalic height along with aerobic endurance and the index of swimming explained approximately 82.4% of the performance of youth competitive swimmers (Saavedra et al., 2010).

Regarding body composition, the % body fat, fat free mass, and arm muscle area correlated positively and significantly with the propulsive force of the arm. The fat mass and lean mass both seem to contribute to the performance of swimmers (Jurimae et al., 2007; Latt et al., 2009b; Saavedra et al., 2010). Swimming does not seem to favour large gains in muscle mass because such gains would reduce floatability and impair performance (Benefice and Ndiaye, 2005; Santos et al., 2007). Avlonitou et al. (1997) studied the effects of competitive swimming on body composition, and they observed that the bone density and lean body mass in the lower limbs were not affected by swim training, but there was a decrease in body fat. Hypothetically, this response can be related to the reduction in protein catabolism and the reduced fat formation due to the effects of IGF-1, which are reflected in the body composition and physical performance (Hulthen et al., 2001).

The AMA has been considered an important variable for swimmers due to its association with the propulsive force capacity. In a previous study we observed a relationship between the propulsive force of the arm and the AMA (Santos et al., 2012). In addition, the AMA changes in response to strength training, what improves sprint swimming times (Dos Santos et al., 2013).

The multiple linear regression models showed that % body fat and body height were positively and significantly correlated with the propulsive force of the arm, predicting 22% of the variation in this variable, regardless of the stage of biological maturation. Body composition can be influenced by environmental factors (*e.g.,* diet and exercise) and by genetic and hormonal factors. From an endocrine perspective, the levels of secreted steroid hormones, which are responsible for somatic and physiological changes during the maturation process, may also be influenced by factors such as the intensity of training and nutrition. However, it is during puberty that the greatest changes in body composition occur due to increases in muscle mass (Hulthen et al., 2001).

Landaeta-Jiménez et al. (2008) analyzed the physical growth and body composition of 178 swimmers (114 males and 64 females) aged between 7 and 18 years. Swimmers had a larger body size but less fat compared to reference values; however, the difference was smaller with increased growth and the maturity level. This result seems to be explained by an increase in the levels of male hormones that generate the marked development of muscle mass during more advanced maturation stages (Hansen et al., 1999). The mechanism that explains these changes might be related to the endocrine and paracrine effects of agents such as growth hormone (GH) and IGF-I (insulin-like growth factor), of which actions promote anabolic muscle protein synthesis and stimulate the hypertrophy of muscle fibres (Hulthen et al., 2001).

Notably, although the maturation processes during pre-puberty and puberty are independent, indicators of sexual and somatic maturation are positively correlated, suggesting that an individual with advanced/delayed sexual maturation will have an advanced/delayed increase in body height (Beunen et al., 2002). Thus, it appears that the different stages of biological maturation (i.e., the age at which various percentages of individuals reach adult body height, the age at which different stages of skeletal maturation are achieved and the age of peak height velocity), can occur together and next to one another (Silva et al., 2004). Indeed, the interdependence of the somatic changes (body height and % body fat) that occur during the course of physical growth during transition from childhood to adolescence may explain, at least in part, the performance results and greater capacity to produce propulsive forces during swimming (Latt et al., 2009b; Matthys et al., 2012). Understanding these associations may contribute to the selection of youth swimmers and to performance improvement in activities requiring greater force production. Since propulsive force is highly correlated with performance in swimming, intervention studies are necessary to investigate the potential effects of strength training on the propulsive force of the arm.

## Conclusion

The propulsive force of the arm is positively associated with body composition and anthropometric indicators in youth swimmers. Body height and body fat content are related to propulsive force of the arm after controlling for the maturation stage.

## Figures and Tables

**Table 1:** Descriptive analysis (mean ± standard-deviations, minimum and maximum value) of anthropometric characteristics, body composition and propulsive force of the arm (PFA).

	**Minimum (n=56)**	**Maximum (n=56)**	**X̄± SD (n=56)**
Age (years)	9.0	17.0	14.4 ± 1.8
Sexual Maturation	2.0	5.0	3.4 ± 0.8
***Anthropometry***			
Body mass (kg)	28.3	80.7	55.8 ± 11. 2
Body height (cm)	134.0	184.5	167.1 ± 11.6
Arm span (cm)	65.5	193.5	170.3 ± 23.4
Sitting height (cm)	54.0	143.0	86.8 ± 11.1
AMA (cm^2^)	19.9	58.2	36.1±8.7
***Body Composition***			
Triceps skinfold (mm)	4.0	20.0	10.2 ± 3.5
Subscapular skinfold (mm)	5.0	17.0	9.4 ± 2.7
Body Fat (%)	8.9	29.7	18.1 ± 4.6
Fat-mass (kg)	2.8	21.2	10.3 ± 3.9
Fat-Free mass (kg)	25.4	59.4	45.4 ± 8.3
***Propulsive force***			
PFA (kgf)	5.7	39.1	17.5 ± 8.5

**Table 2:** Linear regression coefficients (beta estimates, 95% confidence intervals, and p-values), showing estimated change in propulsive force of the arm by the maturation stage

	**Propulsive force of arm (kgf)**			

	**N**	**β**	**95% CI**	**p**
Maturation stage (Tanner: 2–5) +		**-**	**-**	**-**
% body fat +	56	0.318	0.091 a 1.067	**0.021**
Body Height (cm)	56	0.355	0.036 a 0.491	**0.024**
